# Different color regulation mechanism in willow barks determined using integrated metabolomics and transcriptomics analyses

**DOI:** 10.1186/s12870-022-03909-x

**Published:** 2022-11-15

**Authors:** Jie Zhou, Jiahui Guo, Qingsheng Chen, Baosong Wang, Xudong He, Qiang Zhuge, Pu Wang

**Affiliations:** 1 Jiangsu Academy of Forestry, Nanjing city, China; 2 Nanjing Forestry University, Nanjing city, China

**Keywords:** Salix, Bark color, Metabolomics, Transcriptome, Anthocyanin, Carotenoid

## Abstract

**Background:**

The rich yellow-orange to vividly deep red bark of willow (*Salix* spp.) branches have high ornamental and economic value. However, the mechanism underlying the regulation of willow branch color remains unknown. Therefore, we performed metabolomics and transcriptomics analyses of purple, green, and red willow barks to elucidating the mechanisms regulating color development.

**Results:**

Seven anthocyanins were isolated; pelargonidin, petunidin 3-O-rutinoside, and cyanin chloride were the most abundant in red bark, whereas pelargonin chloride was most abundant in purple bark. The green bark contained the highest level of malvidin; however, the malvidin level was not significantly higher than in the red bark. The purple bark contained the largest amount of canthaxanthin, a carotenoid pigment. The integrated pathways of flavonoid biosynthesis, carotenoid biosynthesis, and porphyrin and chlorophyll metabolism were constructed for the willow barks. Among the three barks, the expression of the structural genes *ANS*, *ANR*, and *BZ1*, which are involved in anthocyanin synthesis, was the highest in red bark, likely causing anthocyanin accumulation. The expression of *CrtZ*, which participates in the carotenoid pathway, was the highest in purple bark, likely leading to canthaxanthin accumulation. The high expression of *DVR*, *POR*, and *CRD1* may be associated with green pigment synthesis in the chlorophyll biosynthesis pathway.

**Conclusions:**

Purple bark color is co-regulated by anthocyanins and carotenoids, whereas red bark is characterized by anthocyanin accumulation and chlorophyll degradation. The green pigment is regulated by maintaining chlorophyll synthesis. *BZ1* and *CrtZ* are candidate genes regulating anthocyanin and canthaxanthin accumulation in red and purple barks respectively. Collectively, our results may facilitate the genetic breeding and cultivation of colorful willows with improved color and luster.

**Supplementary Information:**

The online version contains supplementary material available at 10.1186/s12870-022-03909-x.

## Background

Willows, belonging to the genus *Salix* of the Salicaceae family, are important landscape trees in China. The characteristics of willow, including their variable forms, branching morphology, and seasonally changing colors, have made them increasingly popular in gardening, horticulture, and landscape architecture [1]. Willow, particularly the weeping willow (*Salix babylonica*), has been cultivated for thousands of years. In the past 20 years, the golden weeping willow with bright golden branches in winter has increased in popularity. The colors of willow bark are diverse, including green, purple, yellow, white, brown, and red [2]. In recent years, the sale of colorful branches has thrived well in the landscape industry because of their novelty, leading to considerable economic profits. Therefore, the production of colorful willow branches is the primary focus of willow breeding. Although this variation is rich in hybrid generations, the underlying genetic mechanism is not well-understood.

The secondary metabolites determining color development in plants include three classes of compounds as follows: flavonoids, betalains, and carotenoids [3, 4]. Flavonoids, which include anthocyanins, produce pale yellow, red, violet, and purple to blue colors in flowers, seeds, and fruit skin [5]. The six most common anthocyanins that accumulate in flowers and fruits are cyanidin, petunidin, delphinidin, peonidin, pelargonin, and malvidin [6]. Accordingly, these compounds can change the color of plants from pink to blue-violet [7, 8]. Peonidin and cyanidin result in purple to red color in the skin, whereas pelargonin is responsible for brick red-colored skin. The presence of delphinidin, malvidin, and petunidin results in blue and purple colors. Betalains, which originate from tyrosine, are found in plants of the order Caryophyllales and in beetroot, where they produce yellow-to-red coloring [9]. Xanthophylls are the main carotenoids endowing flower petals and fruits with a pale to deep yellow color [10–12]. Combinations of flavonoids and carotenoids in the same organ increases color variety.

The flavonoid biosynthesis pathway has been well-characterized in many plants, including *Arabidopsis* [13], yam [14], and grapevine [15]. Flavonoids are not only synthesized as defense metabolites in response to abiotic stress in trees, such as poplars and ginkgo [16, 17], but also affect fruit quality and nutrition, such as in tomato [18] and kiwi fruit [19]. Anthocyanins, as a subgroup of flavonoids, render color to plants by modifying the anthocyanidins with sugars and acyl acids. The synthesis of precursors is catalyzed by the enzymes chalcone isomerase (CHI), flavonoid 3′, 5′-hydroxylase (F3′5′H), chalcone synthase (CHS), and flavonoid 3′-hydroxylase (F3′H). Subsequently, dihydrokaempferol, dihydroquercetin, and dihydromyricetin are converted into colorless anthocyanins. The catalytic function of dihydroflavonol 4-reductase (DFR) and anthocyanidin synthase (ANS) results in the production of anthocyanidin [20, 21]. Anthocyanins are the pigments generated by this complex pathway through glycosylation reactions catalyzed by UDP-glucosyltransferase and glutathione *S*-transferase [22].

Chlorophyll is a fat-soluble green pigment involved in photosynthesis in plants. Chlorophyll and heme have the same primary biosynthesis steps, beginning at 5-aminolaevulinic acid and then branching from protoporphyrin IX catalyzed by the enzyme porphobilinogen deaminase (HEMC, HEMD, HEME, HEMF, and HEMG). Subsequently, protoporphyrin IX combines with Mg^2+^ to form Mg-protoporphyrin IX through Mg-chelatases CHLH, CHLI1, CHLI2, or CHLD. The catalytic action of the enzyme encoded by divinyl chlorophyllide a 8-vinyl-reductase (*DVR*) generates protochlorophyllide (Pchlide), which is converted to chlorophyllide a. Finally, chlorophyllide a is catalyzed by CHLG-producing chlorophyll a or converted to chlorophyll b by chlorophyllide a oxygenase (CAO) [23]. Carotenoids are among the most widely distributed colorants in flowers and fruits and are responsible for red, orange, and yellow colors [24]. Geranyl–geranyl pyrophosphate is synthesized by the enzyme geranyl–geranyl pyrophosphate synthase, after which phytoene is produced by phytoene synthase (CrtB). The key enzymes are carotenoid cleavage dioxygenases4, ζ-carotene desaturase (ZDS), β-carotene hydrolase, nine *cis*-epoxycarotenoid dioxygenases (NCED), phytoene dehydrogenase (PDS), and zeaxanthin epoxidase (ZEP). The products of each of these enzymes contribute to coloration [25].

The color variation in willow bark is rich, ranging from green and yellow to red. Red bark is rare in southern China; thus, studying the components and regulation of genes that influence the color of willow bark is essential for breeding. In this study, green, purple, and red willow bark were evaluated using metabolome and transcriptome profiling. Metabolites and genes associated with pigment formation in the flavonoid and carotenoid biosynthesis pathways were verified. Our results provide new resources for studying color development in willow bark and a basis for precise breeding to obtain desired willow bark colors.

## Results

### Metabolic analysis of colored willow barks

**Fig. 1:**
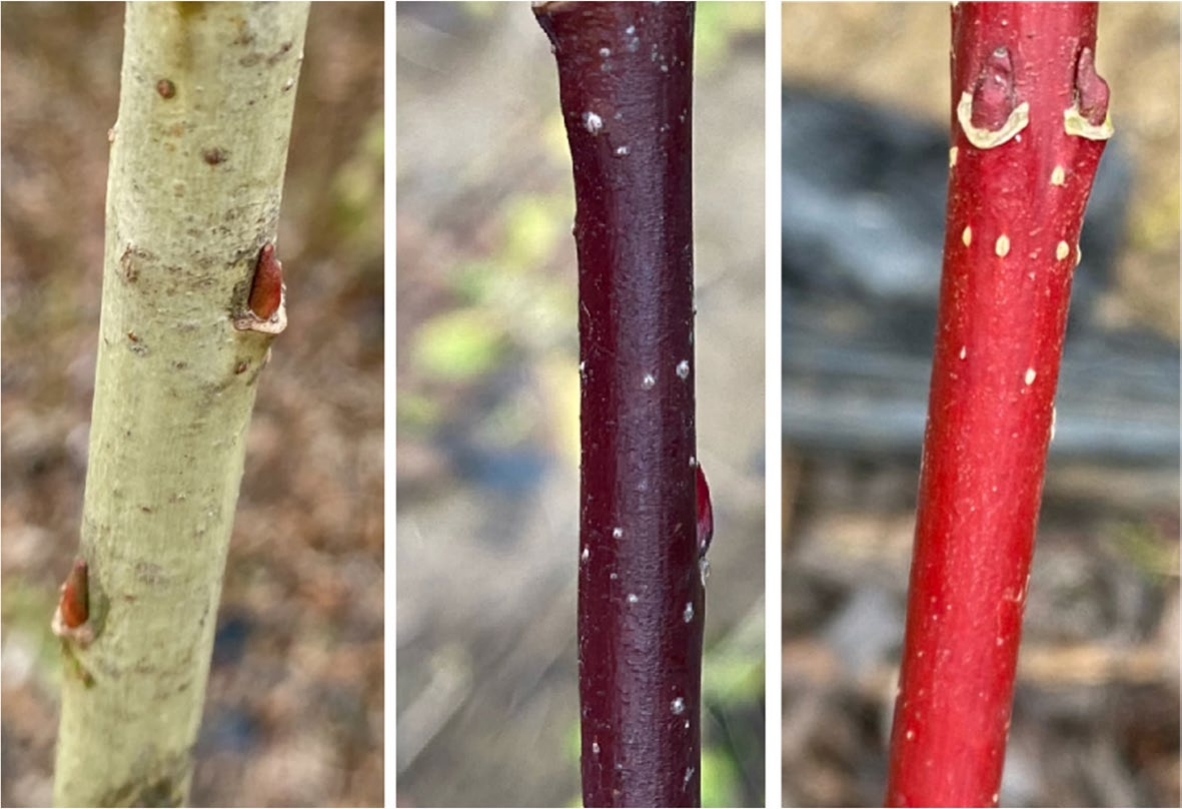
Images of the green, purple, and red willow barks

**Fig. 2:**
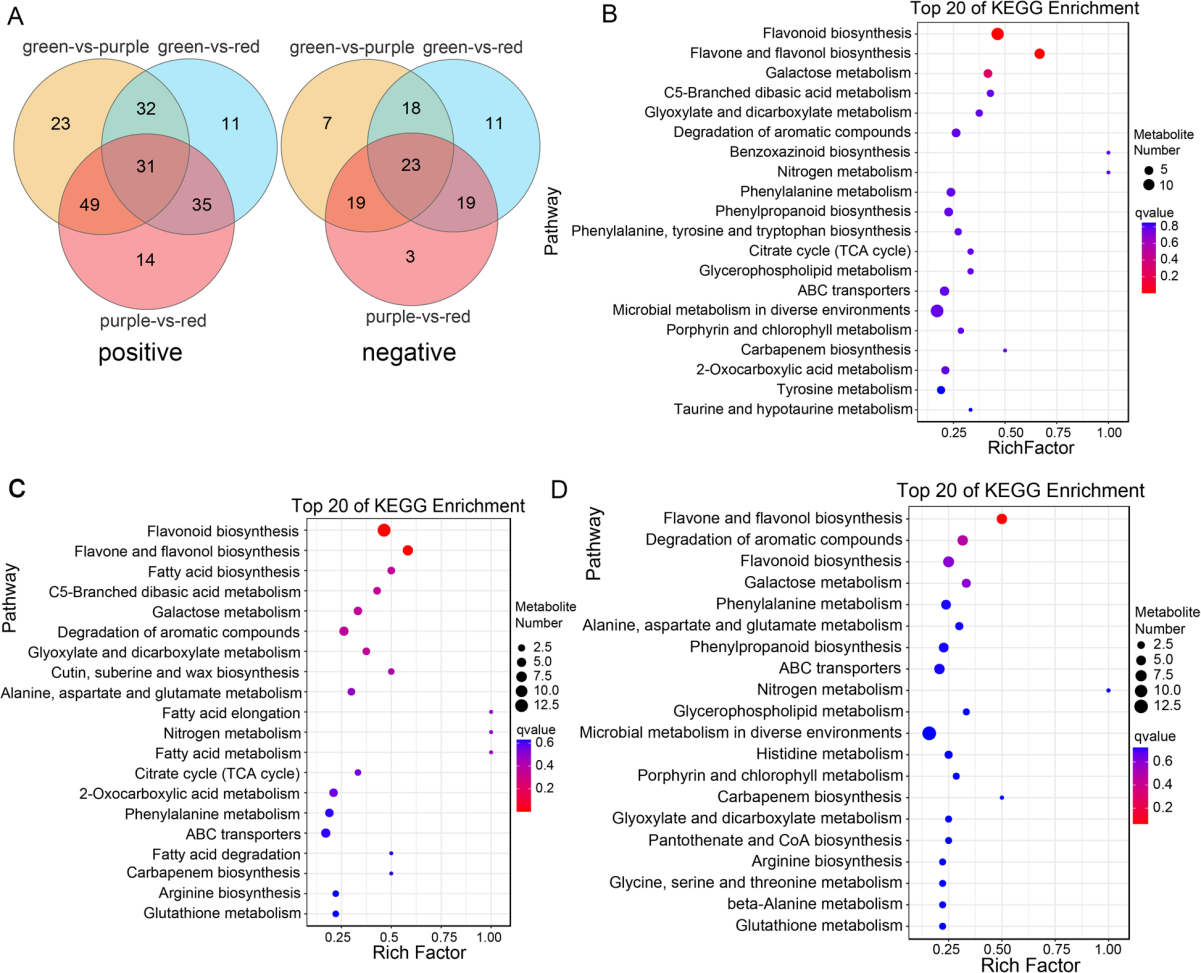
Analysis of differentially accumulated metabolites (DAMs) in willow bark. **A** Venn diagram of DAMs in green vs. purple, green vs. red, and purple vs. red bark comparisons in positive (left) and negative ion modes (right). KEGG pathway orthology of enriched DAMs in **B** green vs. purple bark, **C** green vs. red bark, and **D** purple vs. red bark

The metabolic components of green (G), purple (P), and red (R) willow branch barks were detected and analyzed (Fig. 1). We identified 1639 and 1026 metabolites in positive and negative modes, respectively (Table S1). In the positive mode, 23, 11, and 14 differential metabolites were identified in the three comparisons, whereas in the negative mode, there were seven, 11, and three differential metabolites (Fig. 2a). In the G vs. P, G vs. R, and P vs. R comparisons, 135, 109, and 129 metabolites were detected, of which 55 (40.7%), 68 (62.4%), and 70 (54.3%) were upregulated, respectively (Table S2). To verify the function of the differentially accumulated metabolites (DAMs), Kyoto Encyclopedia of Genes and Genomes (KEGG) pathway enrichment was conducted to annotate the DAMs. Metabolites of flavonoid biosynthesis (ko00941) and flavone and flavonol biosynthesis (ko00944) were enriched in the three groups of G vs. P (Fig. 2B), G vs. R (Fig. 2C), and P vs. R (Fig. 2D). In flavone and flavonol biosynthesis pathway, there were eight, seven, and six enriched DAMs in the G vs. P, G vs. R, and P vs. R groups, respectively; in flavonoid biosynthesis, there were 13, 13, and seven metabolites in the G vs. P, G vs. R, and P vs. R groups, respectively. Porphyrin and chlorophyll metabolism pathway (ko00860) was enriched in groups G vs. P (Fig. 2B) and P vs. R (Fig. 2D). There were two, one, and two metabolites in the G vs. P, G vs. R, and P vs. R groups, respectively.

### Identification of DAMs involved in flavonoid biosynthesis, carotenoid biosynthesis, and porphyrin and chlorophyll metabolism pathways

**Fig. 3:**
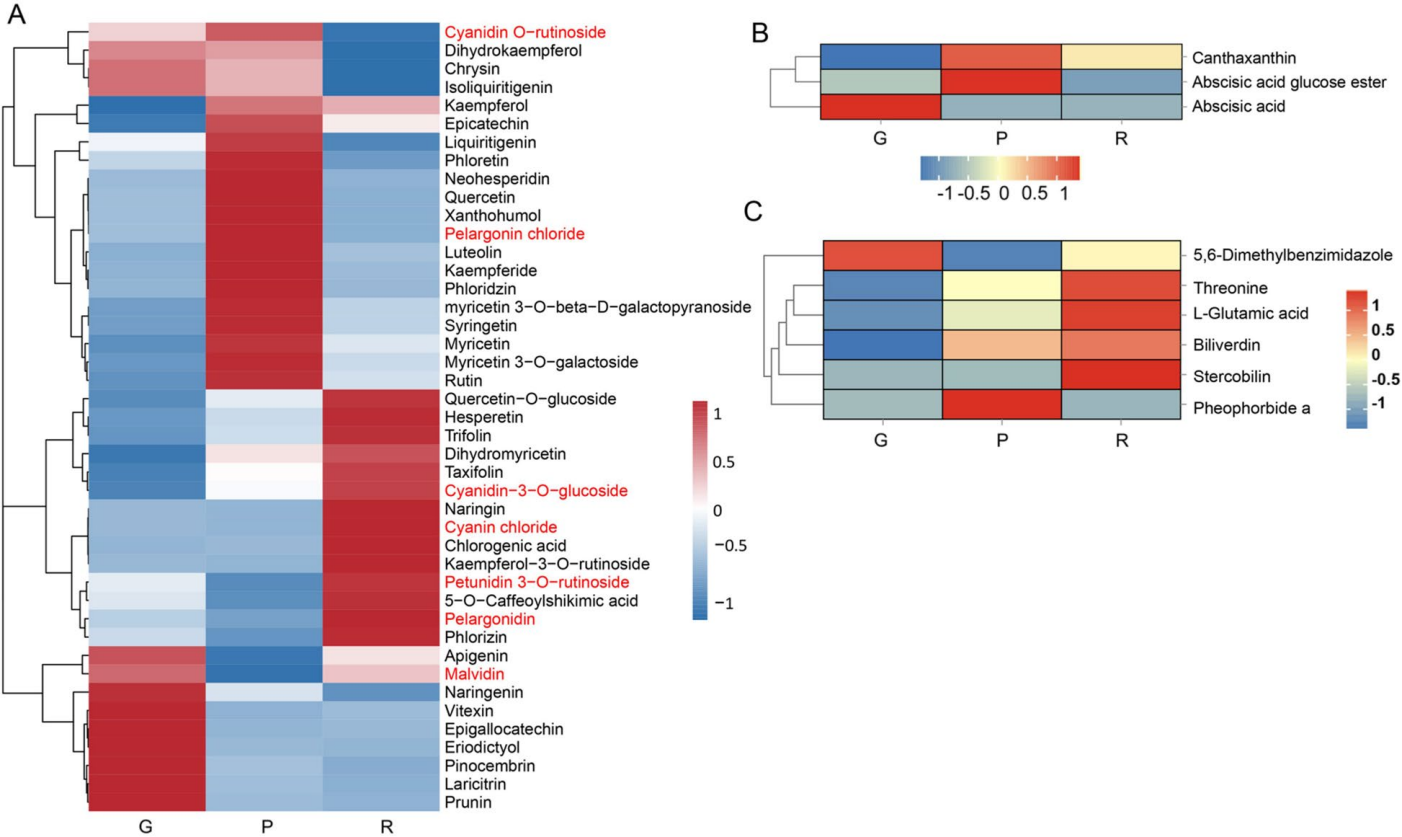
Heatmap of DAMs involved in flavonoid and carotenoid biosynthesis pathways. **A** Heatmap of DAMs involved in flavonoid biosynthesis pathway. The red DAMs are anthocyanin. **B** Heatmap of DAMs in carotenoid biosynthesis pathway. **C** Heatmap of DAMs in porphyrin and chlorophyll metabolism pathway. R, red bark; P, purple bark; G, green bark

Differences in the accumulation of metabolites involved in flavonoid, anthocyanins, and flavone and flavonol biosynthesis are illustrated as a heatmap (Fig. 3). A total of 43 flavonoids were identified and quantified: 14 were present in large amounts in red bark, 15 in purple bark, and nine in green bark. The contents of quercetin, kaempferide, phloretin, neohesperidin, xanthohumol, luteolin, phloridzin, syringetin, rutin, myricetin-3-O-galactoside, and myricetin were the highest in purple willow bark among the three barks. Naringenin, vitexin, epigallocatechin, eriodictyol, pinocembrin, laricitrin, and prunin levels were the highest in green bark. Trifolin levels increased from green to purple and then red bark. Seven anthocyanins were detected, among which pelargonidin, petunidin 3-O-rutinoside, and cyanin chloride were the most abundant in red bark. Pelargonin chloride was the most abundant in purple bark. The level of malvidin was the highest in the green bark. These results suggest that anthocyanins regulate the production of pigment molecules in willow barks. Three compounds, canthaxanthin, abscisic acid glucose ester, and abscisic acid, were detected in carotenoid biosynthesis pathway (Fig. 3B). Six metabolites of porphyrin and the chlorophyll metabolic pathway were identified (Fig. 3C).

### Transcriptome profile characterization

**Fig. 4:**
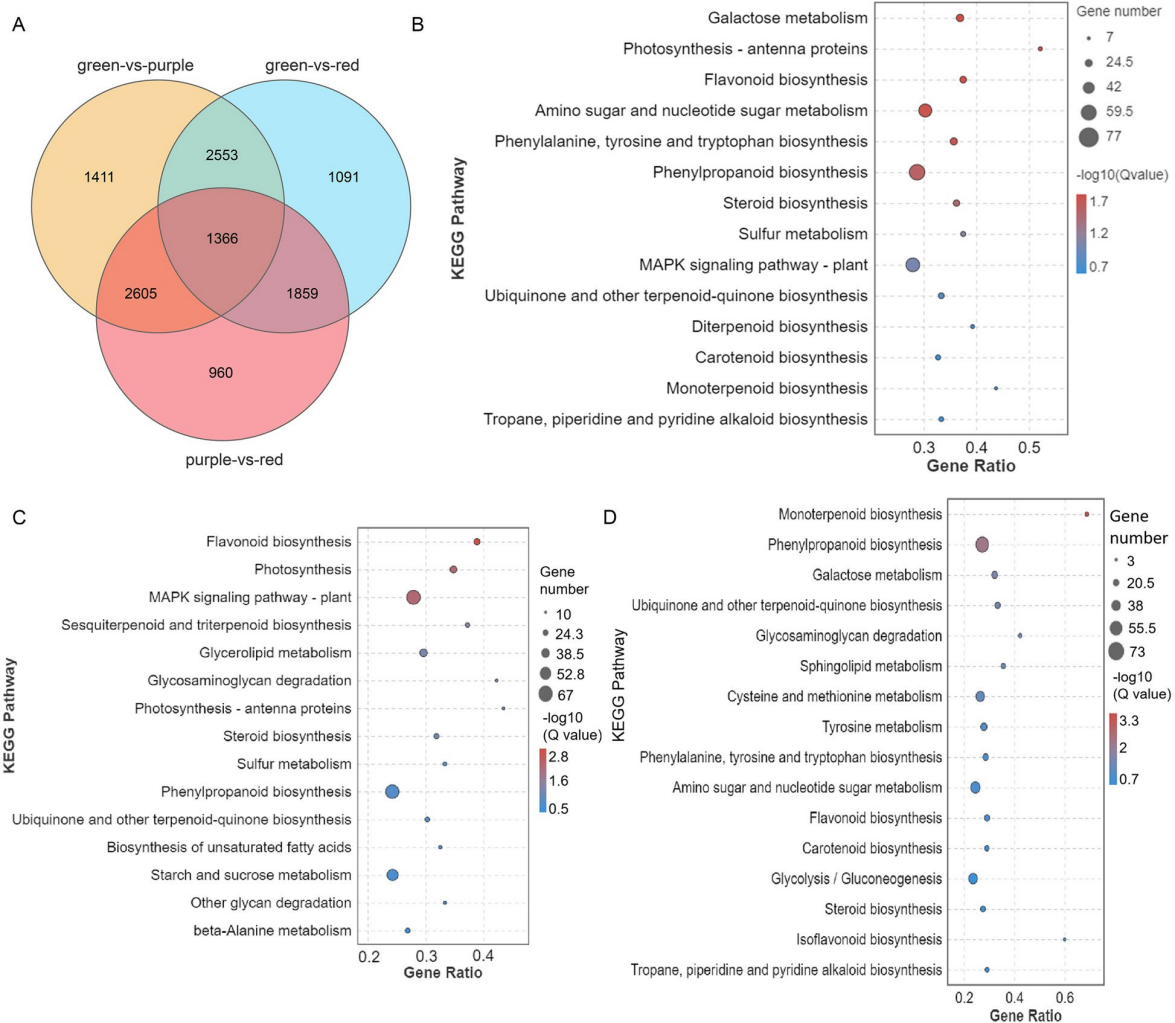
Characterization of transcriptome profiling. **A** Venn diagram of differentially expressed genes (DEGs) among the three colored bark comparison groups. **B**–**D** Enrichment bubble diagram of DEG–KEGG metabolic pathway analysis for (**B**) green vs. purple, (**C**) green vs. red, and (**D**) purple vs. red bark

To investigate the putative genes regulating willow bark color, transcriptome sequencing was conducted. A total of 448,839,796 raw reads were obtained with a Q30 > 93.15% (Table S3). After annotation, 7935, 6896, and 6790 differentially expressed genes (DEGs) were annotated in the G vs. P, G vs. R, and P vs. R comparison groups, including 4519, 3360, and 2705 upregulated genes and 3416, 3509, and 4085 downregulated genes, respectively (Table S4). In KEGG pathway functional analysis, we focused on pigment biosynthesis-associated pathways. Flavonoid biosynthesis (ko00941) was significantly enriched in the three group comparisons, with 27, 28, and 21 genes enriched in the G vs. P, G vs. R, and P vs. R groups, respectively (Fig. 4). Carotenoid biosynthesis was significantly enriched in the G vs. P and P vs. R groups but not in the G vs. R group (Fig. 4B–D). Isoflavonoid biosynthesis was enriched in P vs. R (Fig. 4D).

### Real-time quantitative polymerase chain reaction validation of DEGs in flavonoid and carotenoid biosynthesis pathways

**Fig. 5:**
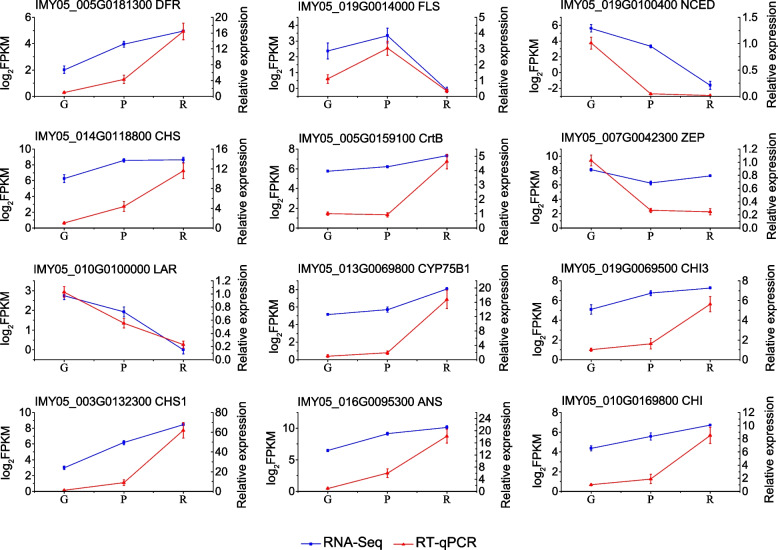
The relative expression level between RT-qPCR and RNA-seq data of genes involved in flavonoid and carotenoid biosynthesis. The relative expression value was calculated using the 2^−ΔΔCt^ method with β-actin gene of *Salix* as internal control. Error bars represent the mean ± SD of three biological replicates (*n* = 3). FRKM, fragments per kilobase of exon per million mapped fragments; G, green bark; P, purple bark; R, red bark

To verify the quality of the transcriptome sequencing results and genes involved in flavonoid and carotenoid biosynthesis, 12 genes (*DFR*, *FLS*, *ANS*, *CHS*, *CHI*, *LAR*, *CYP75B1*, *CHI3*, *CHS1*, *CrtB*, *ZEP*, and *NCED*) were evaluated using real-time quantitative polymerase chain reaction (RT-qPCR). The gene expression trends determined using RNA-sequencing (RNA-seq) were the same as those determined using RT-qPCR for the G, P, and R bark samples (Fig. 5). These results demonstrate that the sequencing transcriptome data are reliable.

### Integration analysis of metabolites and transcripts in the flavonoid biosynthesis pathway

**Fig. 6:**
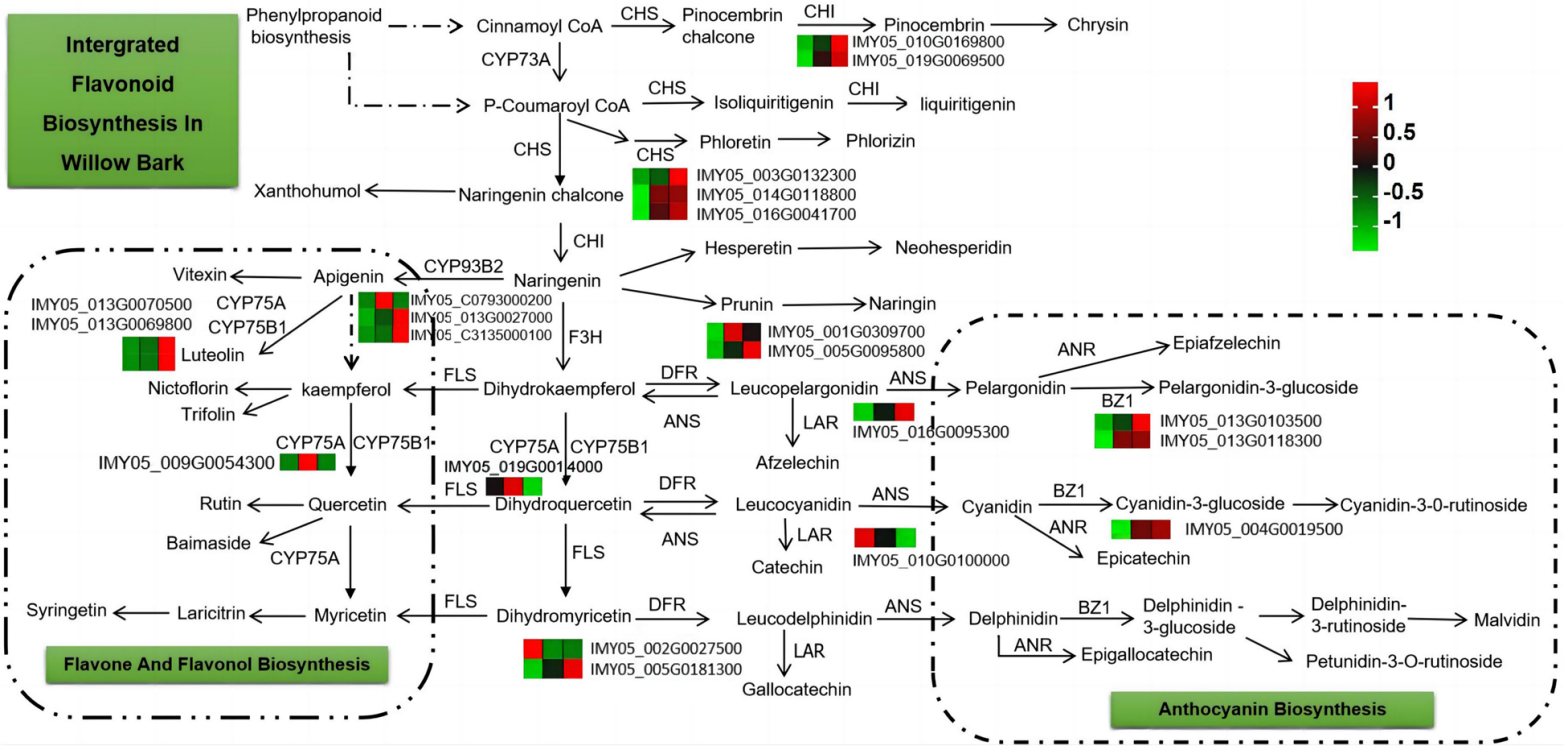
Integrated analysis of flavonoid biosynthesis [26] based on metabolite and transcript analyses. The heatmap of the genes refers to the Z-score normalization of FPKM value of the samples, and the color scale from minimum (green) to maximum (red) corresponds with low to high gene expression, respectively. The three samples refers to the average value of the three groups (*n* = 3 each) for green bark (G), purple bark (P), and red bark (R); each sample was analyzed in biological triplicates. *CHS*, chalcone synthase; *ANR*, anthocyanidin reductase; *ANS*, anthocyanidin synthase; *DFR*, dihydroflavonol 4-reductase; *CHI*, chalcone flavanone isomerase; *BZ1*, bronze-1; *LAR*, leucoanthocyanidin reductase; *FLS*, flavonol synthase; *CYP75A*, flavonoid 3', 5'-hydroxylase; *CYP75B1*, flavonoid 3'-monooxygenase; *CYP93B2*, cytochrome P450 93B2; *CYP73A*, trans-cinnamate 4-monooxygenase; and *F3H*, flavanone 3-hydroxylase

Integration of the results illustrating the changes in metabolite and transcript levels can improve the understanding of the regulatory pathway(s) mediating color development in willow bark. The flavonoid biosynthesis and flavone and flavonol biosynthesis pathways were significantly enriched in the three groups of bark color. As shown in Fig. 6, the gene expression levels of *CHI*, *ANS*, and *BZ1* were the highest in the red bark. The expression of *FLS* was higher in the purple bark than in the other two barks. The expression levels of the downstream regulatory genes *ANR* and *ANS* were consistent with the contents of pelargonidin and epicatechin. In addition, the expression level of *LAR* was high in the green and purple barks, and the epigallocatechin content was highest in the green bark. These results support that different regulatory pathways produce different-colored pigments. Higher levels of pelargonidin, trifolin, phlorizin, and hesperetin accumulated in the red bark than in the purple or green bark (Fig. 6). Higher levels of quercetin, rutin, syringetin, myricetin, neohesperidin, phloretin, and xanthohumol were detected in the purple bark than in the green or red bark. The expression of *CYP93B2* (IMYO5_C0793000200), *CYP75A* (IMYO5_009G0054300), *FLS* (IMYO5_019G0014000), and *F3H* (IMYO5_001G0309700) was consistent with the metabolite contents of luteolin, rutin, quercetin, myricetin, syringetin, neohesperidin, and phloretin in the purple bark. Thus, these genes may be involved in the accumulation of flavonoids in the purple bark. The highest expression of *CHI* ((IMYO5_016G0095300), *CHS* (IMYO5_003G0132300), ANS (IMYO5_016G0095300), *BZ1* (IMYO5_013G0103500), and *DFR* (IMYO5_005G0181300) in red bark compared to that in the green and purple bark were consistent with the contents of phlorizin, hesperetin, naringin, cyanin chloride, cyanidin-3-O-glucoside, dihydromyricetin, pelargonidin, and petunidin-3-O-rutinoside.

### Reconstruction of carotenoid biosynthesis and porphyrin and chlorophyll metabolic pathways for willow barks

**Fig. 7:**
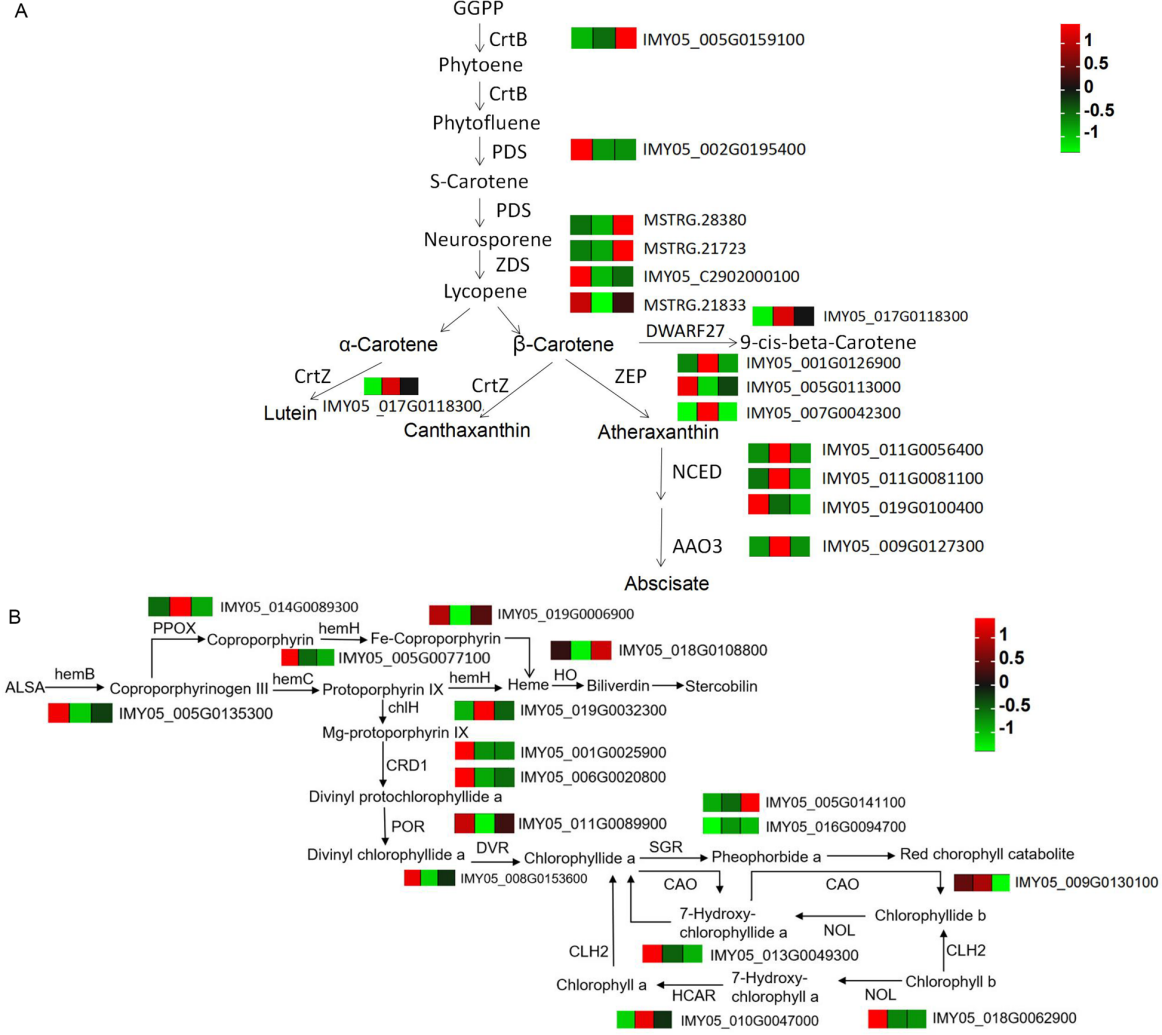
Integrated analysis of carotenoid biosynthesis and porphyrin and chlorophyll metabolism pathways in willow barks. **A** Reconstruction of the carotenoid biosynthesis pathway [27]. **B** Reconstruction of the porphyrin and chlorophyll metabolism pathway [28]. The gene heatmap refers to the z-score normalization of the FPKM value of the samples, and the color scale from minimum (green) to maximum (red) corresponds with low to high gene expression, respectively. The three samples in order are G (green), P (purple), and R (red) bark. Each sample was analyzed in biological triplicates. Genes are defined as follows: *CrtZ*, beta-carotene 3-hydroxylase; *CrtB*, phytoene synthase; *AAO3*, abscisic aldehyde oxidase; *ZDS*, ζ-carotene desaturase; *PDS*, phytoene dehydrogenase; *ZEP*, zeaxanthin epoxidase; *NCED*, 9-cis-epoxycarotenoid dioxygenase; *GGPP*, geranylgeranyl pyrophosphate synthase; *CRD1*, magnesium-protoporphyrin IX monomethyl ester [oxidative] cyclase; *hemH*, ferrochelatase; *HO*, heme oxygenase; *hemC*, porphobilinogen deaminase; *POR*, NADPH-cytochrome P450 reductase; *hemB*, delta-aminolevulinic acid dehydratase; *DVR*, divinyl chlorophyllide a 8-vinyl-reductase; *HCA*R, 7-hydroxymethyl chlorophyll a reductase; *CHLI*, magnesium chelatase subunit chlI; *CAO*, chlorophyllide a oxygenase; *SGR*, stay green; *PPOX*, protoporphyrinogen oxidase; *chlH*, magnesium chelatase*; NOL*, chlorophyll(ide) b reductase; *CLH2*, chlorophyllase 2

In the carotenoid biosynthesis pathway, 15 DEGs were annotated, including one *CrtB*, one *PDS*, four *ZDS*, one *DWARF27*, one *CrtZ*, three *ZEP*, three *NCED*, and one *AAO3*, in the colored willow bark samples (Fig. 7A). Four genes showed the highest expression in purple bark: *CrtZ*, two *ZEP*, two *NCED*, and one *AAO3*. The levels of canthaxanthin and abscisic acid glucose ester metabolites were the highest in the purple bark compared to that in the other barks, supporting the role of various genes in the synthesis of carotenoids and their derivatives in different bark colors. In the porphyrin and chlorophyll metabolism pathway, 16 DEGs were enriched, of which one *HCAR*, one *PPOX*, and one *chlH* showed the highest expression in the purple bark (Fig. 7B).

### Correlation between DEGs and DAMs associated with pigment regulation

**Fig. 8:**
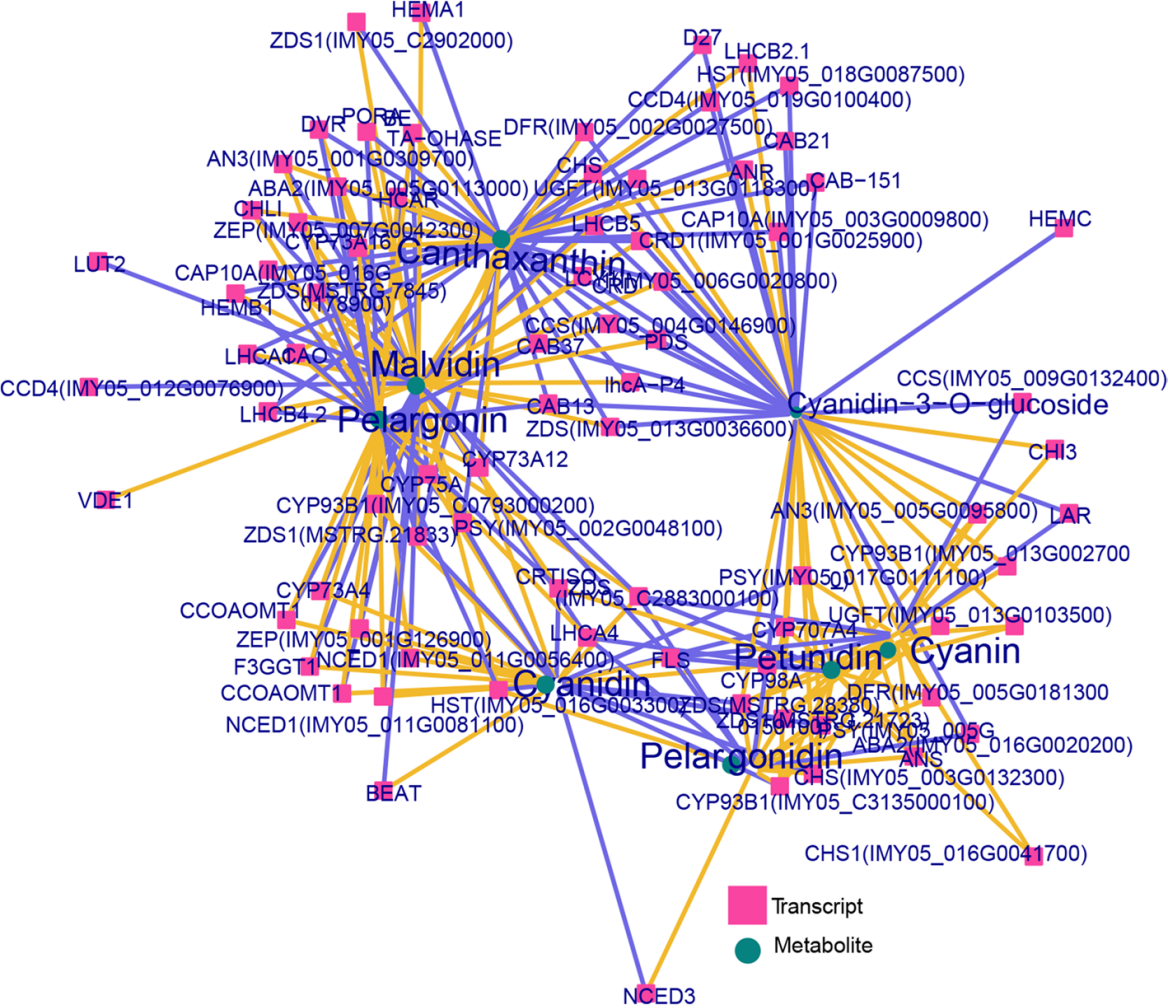
Correlation between differentially expressed genes and metabolites. Yellow line: positive correlation between DEGs and DAMs; blue line: negative correlation between DEGs and DAMs

A correlation network was constructed to investigate the correlation between pigment-associated metabolites and DEGs involved in flavonoid and carotenoid biosynthesis and porphyrin and chlorophyll metabolic pathways (Fig. 8). Malvidin, pelargonin, and cyanidin-3-O-glucoside were associated with numerous genes, indicating that genes influence these components. Cyanidin-3-O-glucoside and canthaxanthin were associated with genes, such as *ANR*, *DFR*, *CHS*, and *PDS*. Malvidin shares the same regulatory genes as canthaxanthin. Pelargonin was the hub metabolite associated with malvidin, canthaxanthin, cyanidin-3-O-glucoside, and petunidin-3-O-rutinoside (Fig. 8).

## Discussion

Integrated metabolome and transcriptome analyses have been increasingly applied in studies of color formation in plants [29]. To our knowledge, this is the first study to perform these analyses to elucidate the regulatory mechanism of willow bark color. Mining of DAMs and DEGs involved in pigmentation in willow bark can provide a fundamental basis for the genetic breeding of colorful willows.

Plant colors are the products of flavonoid, chlorophyll, and carotenoid synthesis [29]. Among the various colors, purple, blue, and red depend on the contents of flavonoids/anthocyanins, including cyanidin and peonidin in common [30]. Chlorophylls present in photosynthetic reaction systems are responsible for green coloration [31], and yellow to orange colors are attributed to carotenoids [32]. However, the regulation of pigment biosynthesis is complex and influenced by the species and environmental conditions and their interactions. In willow bark, four types of anthocyanin derivatives, including cyanidin, pelargonidin, petunidin, and malvidin, were identified. Pelargonidin is an anthocyanin that commonly produces red pigment in plants [33]; in our study, the accumulation of pelargonidin was correlated with red color in willow bark. Cyanidin-3-O-glucoside is the red-brown component of the ligulate flower [34] and the purple pigment encoded by dominant *Pp* alleles in black rice [35]. No significant difference in cyanidin-3-O-glucoside was observed between purple and red willow barks. The stability and types of pigments are influenced by temperature, hydrogen ion concentration (pH), illuminated environment, and anthocyanin structure [36]. Petunidin 3-O-rutinoside can change from dark red to purple and from blue to green and light yellow in a pH-dependent manner, providing a new approach for regulating the red color based on the soil pH. Pelargonidin mostly appeared red, whereas pelargonidin chloride was purple. Cyanin chloride mostly appeared as red, cyanidin O-rutinoside equally exhibited purple and green, and cyanidin-3-O-glucoside contributed to red and purple coloration. Thus, structural differences in the same anthocyanin lead to different colors in willow bark.

Carotenoids are also natural pigments that color vegetables, fruits, and flowers yellow, orange, and red and supplement the flavonoid/anthocyanin content when their levels decline. Canthaxanthin is a ketocarotenoid that accumulates in *Chlorella zofingiensis* [37] but has not yet been detected in plants [38]. The reddish-orange coloration in trout flesh, flamingo feathers, egg yolks, and koi carp skin is attributed to canthaxanthin [39, 40]. Canthaxanthin was the only carotenoid detected in willow bark in this study. Its content in purple bark was 10- and sixfold higher than that in green and red barks. The purple color was likely determined by the type of carotenoid and anthocyanin combinations, and canthaxanthin plays a major role in the purple variety. Color mutations can be caused by damage to the chlorophyll biosynthesis pathway. Stercobilin, an intermediate in the chlorophyll biosynthesis pathway and a brown pigment, was detected in the red bark. In contrast, the levels of L-glutamic acid, an intermediate product, were higher in the red bark than in the green and purple barks.

Stable purple and red barks in willow are rare and have ornamental and economic value. The structural genes involved in color change related to flavonoid biosynthesis are *CYP73A*, *ANS*, *ANR*, *CHI*, *CHS*, *DFR*, and *F3H* [41]. In total, 13 structural genes were identified in the three colors of willow bark. CHS catalyzes the first step of chalcone synthesis, and CHI catalyzes the stereospecific cyclization of chalcone and 6-deoxychalcone to (2S)-liquiritigenin and (2S)-naringenin [42]. The expression of *CHS* and *CHI* may affect the accumulation of anthocyanins as a branch point [43]. One *CHI* (IMY05_010G0169800) and one *CHS* (IMY05_003G0132300) were expressed at high levels in the red bark, whereas *CHI* (IMY05_019G0069500) and two other *CHS* (IMY05_014G0118800, IMY05_016G0041700) were expressed at comparable levels. These genes may play different roles in flavonoid biosynthesis, including the synthesis of compounds such as phloretin (P bark), phlorizin (R bark), rutin (P bark), luteolin (P bark), apigenin (G bark), hesperetin (R bark) and neohesperidin (P bark). A change in DFR enzyme activity may lead to the production of red transgenic gentian flowers by reducing DHK. The low DHK metabolite content and high pelargonidin content indicate a role for DHK in red pigmentation. Enzyme encoded by *ANS* genes can directly convert the substrate of leucoanthocyanidins into colorful anthocyanidins. The activity of *ANS* and *DFR* may result in a high accumulation of pigmentation. The expression of *ANS* and *AN*R were significantly higher in the red barks than in the green and purple barks, which may result in anthocyanin accumulation. *BZ1* encodes the final enzyme that converts UDP-glucose to water-soluble 3-hydroxyl group anthocyanins to achieve a more stable state [44]. *BZ1* and *ANS* are responsible for anthocyanin accumulation in red-skinned pears [45]. The expression level of *ANS* in the red bark was 11-fold higher than that in the green bark. Regarding the two *BZ1* genes, IMY05_013G0103500 in the red bark was 147- and 44-fold higher than that in the green and purple barks, respectively; IMY05_013G0118300, was 27- and 28-fold higher in the red and purple barks than in the green bark, respectively. This indicates that the expression levels of *ANS* and *BZ1* determine red pigment accumulation in willow bark.

In the carotenoid synthesis pathway, most carotenoid pigments are derived from phytoene, which is synthesized in the first reaction of the pathway catalyzed by CrtB. Red lycopene is subsequently synthesized by PDS and ZDS. Lycopene cyclization produces β-carotene, α-carotene, and their derivatives in two branches [46]. In the primary synthesis steps, the expression of *CrtB* is the highest in red bark. Subsequently, one *PDS* and four *ZDS* were highly expressed in the green bark and partially expressed at high levels in the red bark, indicating that branching of the carotenoid synthesis pathway resulted in the production of different products. Only one gene, *CrtZ* (IMY05_017G0118300), was expressed at high levels in the purple bark compared to the other barks. This is consistent with the content of canthaxanthin. Overexpression of the β-carotene 3-hydroxylase gene (*CrtZ*) from *Agrobacterium aurantiacum* resulted in the accumulation of canthaxanthin [47]. The protein DWARF27 (D27) participates in strigolactone biosynthesis, which regulates rice branching [48]. Thus, *D27* is a candidate gene that regulates the branching and phenotype of colorful willows.

**Fig. 9:**
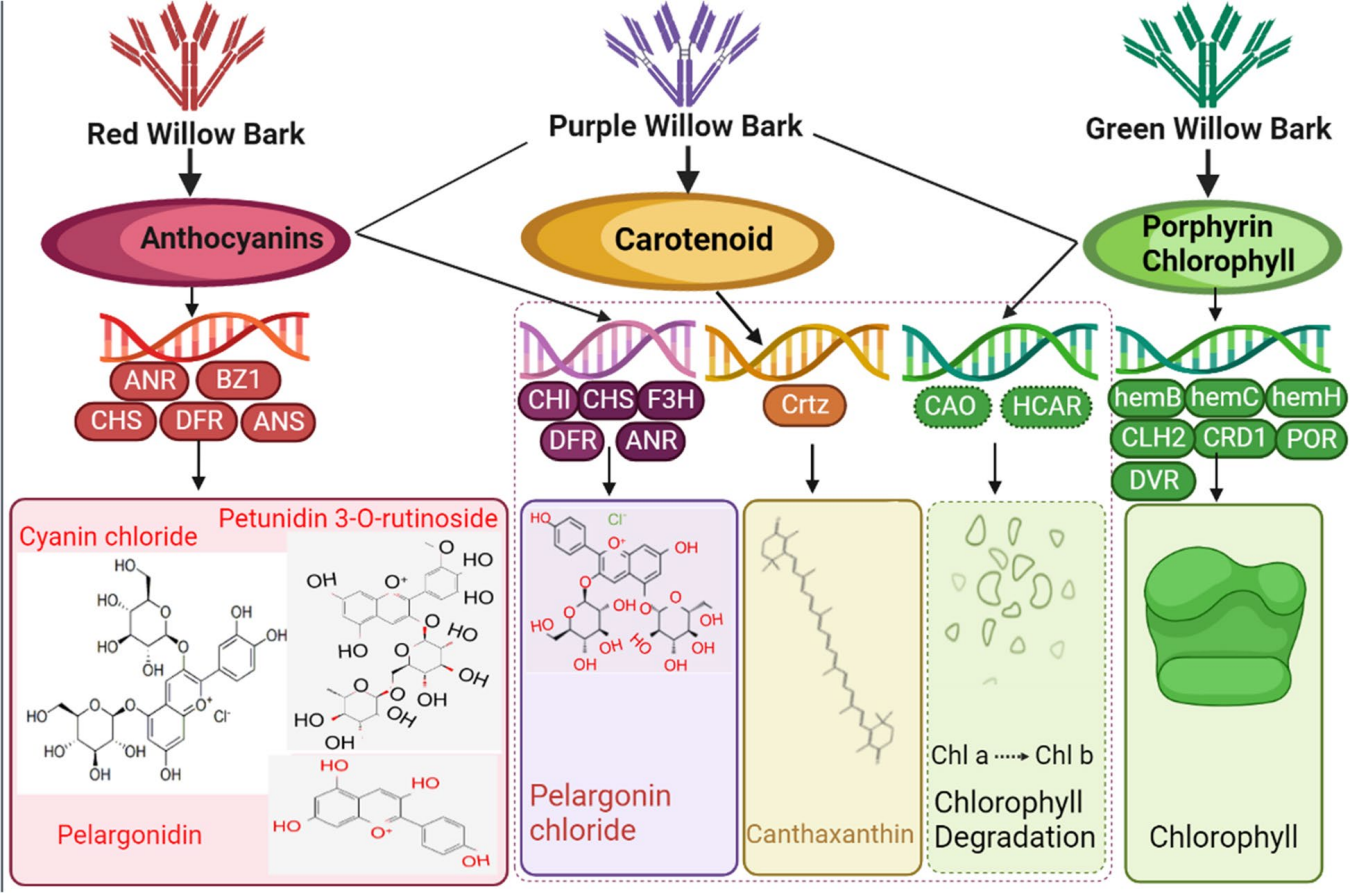
A cartoon illustrating the proposed regulation network of color development in willow barks. *ANR*, anthocyanidin reductase; *BZ1*, bronze-1; *CHS*, chalcone synthase; *DFR*, dihydroflavonol 4-reductase; *ANS*, anthocyanidin synthase; *CHI*, chalcone flavanone isomerase; *F3H*, flavanone 3-hydroxylase; *CrtZ*, beta-carotene 3-hydroxylase; *CAO*, chlorophyllide a oxygenase; *HCAR*, 7-hydroxymethyl chlorophyll a reductase; *hemB*, delta-aminolevulinic acid dehydratase; *hemC*, porphobilinogen deaminase; *hemH*, ferrochelatase; *CLH2*, chlorophyllase 2; *CRD1*, magnesium-protoporphyrin IX monomethyl ester [oxidative] cyclase; *POR*, NADPH-cytochrome P450 reductase; *DVR*, divinyl chlorophyllide a 8-vinyl reductase

The purple color of willow barks is likely conferred by the co-pigmentation interaction of carotenoid and anthocyanin, with carotenoid as a major factor. The expression of most key structural genes involved in the chlorophyll biosynthesis pathway, including *hemH*, *hemB*, *POR*, *CRD1*, *DVR*, and *CLH2*, was the highest in the green bark, indicating that the green color was determined by chlorophyll synthesis. POR is a key enzyme catalyzing the conversion of protochlorophyllide to chlorophyllide in chlorophyll synthesis [49]. CRD1 mainly functions in chloroplast movement. The higher expression of *POR* and *CRD1* in the green bark may explain the maintenance of green pigmentation. Overexpression of the stay-green (SGR) protein in melon led to CHL degradation and leaf yellowing [50]. The highest expression of *SGR* (IMY05_005G0141100) was observed in the red bark, whereas another *SGR* (IMY05_016G0094700) was silenced in the green bark, causing green coloration. Degradation metabolites were detected at high levels in the red bark, indicating that chlorophyll was degraded by the *SGR*. *CAO* is vital for regulating the chlorophyll a/b ratio, and expression of 7-hydroxymethyl chlorophyll a reductase (*HCAR*) may limit chlorophyll b degradation [51]. Overexpression of *HCAR* accelerated chlorophyll degradation in cucumber [52]. Therefore, the highest expression of *CAO* and *HCAR* in the purple bark suggests that chlorophyll degradation occurs via the conversion of chlorophyll a to chlorophyll b. Based on these findings, we constructed a regulatory mode for willow bark coloration (Fig. 9).

## Conclusion

In this study, the pigmentation regulation of bark color in *Salix* was characterized through integrated metabolome and transcriptome analyses. Red bark contained more pelargonidin, petunidin 3-O-rutinoside, and cyanin chloride than purple and green barks. Purple bark contained more pelargonin chloride than red and green barks. Malvidin had the highest level in the green bark; however, there was no significant difference between malvidin levels in green and red bark. Canthaxanthin, a carotenoid metabolite, was detected in purple bark. The structural genes *CHS*, *ANS*, *DFR*, *ANR*, and *BZ1* were annotated in the flavonoid biosynthesis pathway. The high expression of *ANS* and *BZ1* in red bark likely led to anthocyanin accumulation. *CrtZ* exhibited the highest expression level in purple bark, indicating an association with canthaxanthin accumulation. The high expression of *hemH*, *hemB*, *POR*, *CRD1*, *DVR*, and *CLH2* may explain the green pigmentation in the green bark. *SGR* was highly expressed in the red bark and is likely associated with the accumulation of stercobilin in the chlorophyll degradation pathway. *CAO* and *HCAR* expression were the highest in the purple bark, suggesting that chlorophyll degradation occurs through the conversion of chlorophyll a to chlorophyll b and may lead to the production of pheophorbide a. The connection network of anthocyanin and DEGs also suggest that the purple bark is co-regulated by anthocyanins and carotenoids; the red bark is characterized by anthocyanin accumulation and chlorophyll degradation; the green pigment is regulated by maintaining chlorophyll synthesis. Collectively, our results may facilitate the genetic breeding and cultivation of colorful willows with improved color and luster.

## Methods

### Plant materials

The three *Salix* species were collected and deposited in the willow nursery in Dafeng District, Yancheng City, Jiangsu province (33.20–33.35°N, 120.15–120.47°E). This area is in the transition zone between subtropical and warm, humid zones. It is approximately 2 km from the sea, with an altitude of approximately 3 m. The annual average temperature, frost-free period, annual precipitation, and sunshine time are 14.1℃, 213 days, 1042.2 mm, and 2238.9 h, respectively. The species were preserved by the authors who have permission to collect willow barks. The purple bark was from *Salix* × *Jiangsuensis ‘J1053’*, G bark from *S. integra*, and R bark from *S. miyabeana*. The voucher specimens of *S.* × *Jiangsuensis ‘J1053’*, *S. integra*, and *S. miyabeana* were deposited at the herbarium of Jiangsu academy of forestry under voucher numbers J1053, P61, and P76, respectively. They are all shrub willow species. *S.* × *Jiangsuensis ‘J1053’* was hybridized by *S. suchowensis* × *S. dasyclados. S. miyabeana* with red bark is an excellent ornamenta variety selected from numerous clones introduced from the United States. It has strong adaptability, curved stems, and red branches, as well as desirable ornamental characteristics. *S. integra* is a robust parent clone with green bark. The red, green, and purple colors of the barks are evident and stable during the seedling stage. The email of the person coordinating sample collection is zjwin718@126.com.

### Sample preparation

The willow bark was scraped from branches using scalpels in the nursery. The bark of each color was collected from six individuals of the same species. The samples were placed in microcentrifuge tubes, rapidly frozen in liquid nitrogen, and stored at − 80 °C until metabolite and RNA isolation. Six biological repeats were performed to identify the metabolites, and three biological repeats were used to determine the transcripts.

### Metabolite extraction and ultra-high performance liquid chromatography-tandem mass spectrometry analysis

Willow bark powder (100 mg), which was ground in liquid nitrogen, was placed in a microcentrifuge tube, and 500 µL water containing 80% methanol was added. The mixture was resuspended, vortexed, and incubated for 5 min. The powder was centrifuged for 20 min at 1500 × g, 4 °C. The supernatant was transferred to a clean microcentrifuge tube containing liquid chromatography-mass spectrometry (LC–MS)-grade water containing 53% methanol and centrifuged for 20 min. The supernatant was absorbed and analyzed using an LC–MS system [53]. A vanquish ultra-high performance LC system (Thermo Fisher Scientific, Germering, Germany) and Orbitrap Q ExactiveTMHF-X mass spectrometer (Thermo Fisher Scientific, Germering, Germany) were used for ultra-high performance LC-tandem MS detection in Gene Denovo (Gene Denovo Co., Ltd., Guangzhou, China). A Hypesil Gold chromatography column (100 × 2.1 mm, 1.9 μm) was used at a column temperature gradient of 40 °C and flow rate of 0.2 mL/min. Eluent a was composed of 0.1% formic acid in positive mode and 5 mM ammonium acetate in negative mode. Eluent b was composed of methanol in both modes. The negative mode was eluent a, pH 9.0, and eluent b, methanol.

### Identification and annotation of metabolites

Raw data were analyzed using Compound Discoverer 3.1 (CD3.1) for each metabolite quantitation. After peak extraction and peak area quantitation, the molecular formula was predicted based on the peaks of molecular and fragment ions. The molecules were searched against the mzCloud, mzVault, and Masslist databases for relative and accurate quantification of the final metabolites. Metabolite data were analyzed in positive and negative ion modes separately. The principle component analysis (PCA) for the quality check of metabolites and multivariate regression analysis were conducted in R language gmodels (v2.18.1) [54]. In the comparison groups, partial least squares discriminant analysis (PLS-DA) was applied using the R package ropls [55] (http://www.r-project.org/), and orthogonal projection to latent structures-discriminant analysis (OPLS-DA) was applied using R package models (http://www.r-project.org/). Differential metabolites were screened based on the variable importance in the projection (VIP) score of the (O)PLS model with a p-value of the *t*-test < 0.05 and VIP ≥ 1 between the two groups. The abundance of differential metabolites was normalized based on the z-score, and hierarchical clustering was performed using the R package pheatmap [56] between the two groups. The annotation and enrichment of differential metabolites were mapped to the KEGG pathway database.

### Transcriptome sequencing and DEG identification

An Omega plant RNA kit (Omega Bio-Tek, Norcross, GA, USA) was used to extract total RNA, and cDNA libraries were constructed using an NEB#7530 kit (#E7530, New England Biolabs, Ipswich, MA, USA). The library quality was assessed using a DNA 1000 assay kit (#5067–1504, Agilent Technologies, Santa Clara, CA, USA). The library was sequenced on an Illumina HiSeq2500 platform (San Diego, CA, USA). Raw reads were filtered by fastp [57] (version 0.18.0) to obtain high-quality clean reads by removing reads containing more than 10% of unknown nucleotides (N) and more than 50% of low-quality (Q-value ≤ 20) bases. The clean reads were mapped to the *Salix suchowensis* (*Salix suchowensis* (ID 36,318) -Genome-NCBI (nih.gov)) genome using HISAT 2.2.4 [58] with the parameters set as “-rna-strandness RF” and others as a default. The mapped reads were assembled by StringTie v1.3.1 [59, 60]. An FPKM (fragment per kilobase of transcript per million mapped reads) value was calculated by StringTie software to quantify the expression abundance and variations of each transcript. DEGs analysis was performed using DESeq2 software [61] (and by edgeR within a group between two samples). Genes with a false discovery rate (FDR) adjusted P value of < 0.05 (FDR = 5%) and absolute fold change ≥ 2 were considered differentially expressed. Annotation of DEGs and KEGG functional enrichment followed the method described by Zhou et al. (2020) [62]. The abundance of DEGs were normalized based on the z-score and the heatmap was performed using Omicsmart, a dynamic real-time interactive online platform for data analysis (http://www.omicsmart.com).

### Real-time quantitative PCR verification

The RNA used for RT-qPCR was reverse-transcribed using Hifair R III 1st Strand cDNA Synthesis SuperMix gDNA digester plus (Yeasen Biotechnology [Shanghai] Co., Ltd., Shanghai, China). RT-qPCR was performed using Genious 2 × SYBR Green Fast qPCR Mix (ABclonal, Wuhan, China) on a LightCycler® 480 II (Roche, Basel, Switzerland). The two-step cycling procedure was performed at 95 °C, 5 min; 40 cycles at 95 °C, 10 s; 60 °C, 30 s, and 40 cycles at 72 °C for one-point sensing. The melting curve cycle conditions were 95 °C (15 s), 60 °C (60 s), and 95 °C (15 s) to continuously obtain the signal. The expression values were analyzed using the 2^**−ΔΔCt**^ method. The figure was drawn using Origin software. Primers were designed using Primer Premier 5 (Premier Biosoft, Palo Alto, CA, USA) and are listed in Table S5.

### Metabolite and DEG correlation network construction

Pearson correlation coefficients were determined to integrate the transcriptomic and metabolomics correlation network. Gene and metabolite were ranked in descending order of their absolute correlation coefficients. Pairs of genes and metabolites involved in the flavonoid, chlorophyll, and carotenoid biosynthesis pathways (with the absolute pearson correlation > 0.5) were applied for metabolite-transcript network analysis using the igraph package in R software [63].

## Supplementary Information

**Additional file 1:** **TableS1.** Information on all metabolites. **Table S2.** Up- and downregulatedmetabolites. **Table S3.** RNA-sequencing profiles. **Table S4.** Number of up- anddownregulated differentially expressed genes. **Table S5.** The primers designedfor RT-qPCR.

**Additional file 2: TableS6. **The metabolites identified in positive ion mode. 

**Additional file 3: Table S7. **The metabolites identified in negative ionmode. 

**Additional file 4: Table S8. **The differential expressed genes identified inwillow barks.

## Data Availability

The transcriptome sequence data were submitted to GenBank of NCBI (https://www.ncbi.nlm.nih.gov/) under the accession no. SRP375909. The associated BioProject, SRA, and Bio-Sample numbers are as follows: PRJNA839146, SAMN28511960, SAMN28511961, SAMN28511962, SAMN28511963, SAMN28511964, SAMN28511965, SAMN28511966, SAMN28511967, and SAMN28511968. The metabolome data were submitted to Metabolights (https://www.ebi.ac.uk/metabolights/) under the data number MTBLS4820.
