# Supplementary material for: Different color regulation mechanism in willow barks determined using integrated metabolomics and transcriptomics analyses
Source: BMC Plant Biol. 2022 Nov 15;22:530. doi: 10.1186/s12870-022-03909-x (PMC9664647; doi:10.1186/s12870-022-03909-x)
Supplement: Supplementary file 1 — Additional file 1: TableS1. Information on all metabolites. Table S2. Up- and downregulatedmetabolites. Table S3. RNA-sequencing profiles. Table S4. Number of up- anddownregulated differentially expressed genes. Table S5. The primers designedfor RT-qPCR. [file 12870_2022_3909_MOESM1_ESM.docx]

Supplemental material

Table S1: Information of all the metabolites

Table S2: The up and down-regulated metabolites

Table S3: The RNA-seq sequencing profiles

Table S4: The number of up and down-regulated DEGs

Table S5: The primers designed for RT-qPCR

Table S6: The metabolites identified in positive ion mode

Table S7: The metabolites identified in negative ion mode

Table S8: The differential expressed genes identified in willow barks

Table S1. Information of all the metabolites

| type | All | MS2 | MS1 | known(MS1+MS2) | unknown |
| --- | --- | --- | --- | --- | --- |
| POS | 1639 | 1229 | 410 | 1639 | 0 |
| NEG | 1026 | 829 | 197 | 1026 | 0 |

POS: positive ion mode; NEG: negative ion mode; MS2: secondary mass spectrometry；MS1：primary mass spectrometry

Table S2. The up- and down-regulated metabolites

| group | | up | down | total | percent of up-regulated metabolites | |
| --- | --- | --- | --- | --- | --- | --- |
| Green vs Purple. POS | 55 | 80 | 135 | 0.407407407 |  |  |
| Green vs Red. POS | 68 | 41 | 109 | 0.623853211 |  |  |
| Purple vs Red. POS | 70 | 59 | 129 | 0.542635659 |  |  |
| Green vs Purple. NEG | 18 | 49 | 67 | 0.268656716 |  |  |
| Green vs Red. NEG | 55 | 16 | 71 | 0.774647887 |  |  |
| Purple vs Red. NEG | 34 | 30 | 64 | 0.53125 |  |  |

POS: positive ion mode; NEG: negative ion mode.

Table S3. The RNA-seq sequencing profiles

| Sample | RawData(bp) | BF_Q20(%) | BF_Q30(%) | BF_N(%) | BF_GC(%) | CleanData(bp) | AF_Q20(%) | AF_Q30(%) | AF_N(%) | AF_GC(%) |
| --- | --- | --- | --- | --- | --- | --- | --- | --- | --- | --- |
| Green-1 | 8231025000 | 8018155883 (97.41%) | 7671555251 (93.20%) | 699362 (0.01%) | 3674737488 (44.64%) | 8123055282 | 7934548783 (97.68%) | 7597406937 (93.53%) | 72502 (0.00%) | 3619056701 (44.55%) |
| Green-2 | 6195933000 | 6032830082 (97.37%) | 5764300342 (93.03%) | 530963 (0.01%) | 2736204724 (44.16%) | 6111107062 | 5964736067 (97.60%) | 5703382474 (93.33%) | 55191 (0.00%) | 2693589850 (44.08%) |
| Green-3 | 7343046000 | 7162790286 (97.55%) | 6859014613 (93.41%) | 624118 (0.01%) | 3272242377 (44.56%) | 7262463916 | 7099761308 (97.76%) | 6802849047 (93.67%) | 63699 (0.00%) | 3231100422 (44.49%) |
| Purple-1 | 6580368600 | 6416835693 (97.51%) | 6144740865 (93.38%) | 567054 (0.01%) | 2957884700 (44.95%) | 6488676984 | 6343578663 (97.76%) | 6079198706 (93.69%) | 57809 (0.00%) | 2910642192 (44.86%) |
| Purple-2 | 7551423300 | 7354900256 (97.40%) | 7034492441 (93.15%) | 650889 (0.01%) | 3395307039 (44.96%) | 7457635839 | 7283526878 (97.67%) | 6971252092 (93.48%) | 65993 (0.00%) | 3346716278 (44.88%) |
| Purple-3 | 8560477200 | 8343949552 (97.47%) | 7979592846 (93.21%) | 735858 (0.01%) | 3781409320 (44.17%) | 8445250183 | 8252845145 (97.72%) | 7898492461 (93.53%) | 76113 (0.00%) | 3722884972 (44.08%) |
| Red-1 | 6742064400 | 6569371563 (97.44%) | 6279919098 (93.15%) | 590685 (0.01%) | 2973239320 (44.10%) | 6654402856 | 6499153239 (97.67%) | 6216972964 (93.43%) | 60455 (0.00%) | 2928253550 (44.00%) |
| Red-2 | 9060447600 | 8835218831 (97.51%) | 8455983799 (93.33%) | 768674 (0.01%) | 4022638437 (44.40%) | 8961655526 | 8758394561 (97.73%) | 8387524332 (93.59%) | 79520 (0.00%) | 3972173063 (44.32%) |
| Red-3 | 7379517900 | 7193465743 (97.48%) | 6874536682 (93.16%) | 640894 (0.01%) | 3264711633 (44.24%) | 7303843288 | 7133057366 (97.66%) | 6820490524 (93.38%) | 64878 (0.00%) | 3226455932 (44.17%) |

Table S4. The number of up- and down-regulated DEGs

| groups | up-regulated | Down-regulated |
| --- | --- | --- |
| Green-vs-Purple | 4519 | 3416 |
| Green-vs-Red | 3360 | 3509 |
| Purple-vs-Red | 2705 | 4085 |

Table S5 The primers designed for RT-qPCR

| Gene ID | primer name | primer sequence |
| --- | --- | --- |
| β-Actin | F | GACCTTCAATGTGCCTGCAA |
| β-Actin | R | ACCATCACCAGAATCCAGCA |
| IMY05_013G0069800 | F | GGGTAATGTTAGGGAGACGGG |
|  | R | AGTGTGCTTCTCCGATCTAACG |
| IMY05_003G0132300 | F | GCCAGCCCAAGTCCAAAGT |
|  | R | GATGGACGGAGACCCAAAAG |
| IMY05_005G0159100 | F | AACTGGGGTTTCTGCTGCC |
|  | R | CAGACCTCGCCACACCTATTAT |
| IMY05_005G0181300 | F | AGGCTGAAACCCACTTGACTT |
|  | R | CCCCATTGATTGTAGGCTTGA |
| IMY05_014G0118800 | F | TTGGATTCAATGGTAGGGCA |
|  | R | GGAGTTCCAGTCGTTAATACCG |
| IMY05_016G0095300 | F | TAACAGCAAAATGTGTCCCCA |
|  | R | AGGTGCGTGGAGGGAACAA |
| IMY05_019G0014000 | F | GAGGGAGGTGGCAGATAAACTT |
|  | R | GAATGGTGAGGGCGGATAGA |
| IMY05_019G0069500 | F | AGGAAGAAGCCCTGGAAAAGA |
|  | R | TGGTATTGGCAAGGCAGGA |
| IMY05_019G0100400 | F | GGCAACCGACTCTACGCAA |
|  | R | GAAGTCGTGGATGAAAGAGGG |
| IMY05_007G0042300 | F | CCGATGACCAGTTGCGAAG |
|  | R | CACTCCCAACCACAAACGACT |
| IMY05_010G0100000 | F | TGATGCTCTGCCTCCTCTGG |
|  | R | GGGTTCTTCCGATTTTCTTTTC |
| IMY05_010G0169800 | F | TTGCTGCTAAATGGAAGGGC |
|  | R | TGGGATGAGAGGGTGAAAAGA |
| IMY05_013G0027000 | F | TTCCCCAGGACACATTGTTATT |
|  | R | ATGTCCCCGTCAGGTTTTAGA |
